# A native-like bispecific antibody suppresses the inflammatory cytokine response by simultaneously neutralizing tumor necrosis factor-alpha and interleukin-17A

**DOI:** 10.18632/oncotarget.19899

**Published:** 2017-08-03

**Authors:** Tianshu Xu, Tianlei Ying, Lili Wang, Xiaohua Douglas Zhang, Ying Wang, Lishan Kang, Tao Huang, Liang Cheng, Liping Wang, Qi Zhao

**Affiliations:** ^1^ School of Life Science, Jilin University, Changchun, Jilin, China; ^2^ School of Basic Medical Sciences, Fudan University, Shanghai, China; ^3^ Faculty of Health Sciences, University of Macau, Taipa, Macau, China; ^4^ Institute of Chinese Medical Sciences, University of Macau, Taipa, Macau, China; ^5^ Novo Nordisk Research Centre China, Beijing, China

**Keywords:** bispecific antibody, Fc heterodimerization, TNF-α, IL-17, rheumatoid arthritis, Immunology and Microbiology Section, Immune response, Immunity

## Abstract

Anti-tumor necrosis factor (TNF) therapies are successful in the treatment of inflammatory disorders. However, some patients with rheumatoid arthritis (RA) fail to response anti-TNF drugs due to the compensation of other inflammatory signals. In this study, to reduce compensatory responses of interleukin-17A (IL-17A) during TNF-α inhibition, we generated an IgG-like bispecific antibodiy (bsAb) against TNF-α and IL-17A through a combination method of electrostatic Fc pairing and light chain crossover. This bsAb exhibited relatively high stability comparable to natural IgG antibodies, and retained the unaltered affinities to both of two targets. BsAb significantly decreased not only the expression level of neutrophil or Th17 chemokines, but also the secretion of IL-6/IL-8 on fibroblast-like synoviocytes (FLS) from a patient with RA. Meanwhile, TNF-α-mediated cellular cytotoxicity of fibroblasts was neutralized by bsAb. Importantly, we demonstrate that the combined blockade of TNF-α and IL-17A is more efficient than inhibition of either factor alone. Our results suggest the IgG-like anti-TNF-α/IL-17A bispecific molecule overcome the limited therapeutic responses using anti-TNF drugs. It may be a promising therapeutic agent for the treatment of autoimmune diseases.

## INTRODUCTION

Currently, the pathogenesis of autoimmune disorders is not fully understood. Immune dysfunction is involved in complicated genetic and environmental factors [1]. Over the past decade, monoclonal antibody (mAb) drugs succeed in treatment of autoimmune diseases [2]. These mAbs have benefited millions of patients with chronic diseases, such as rheumatoid arthritis (RA), psoriasis and Crohn’s disease, and so on.

Tumor necrosis factor alpha (TNF-α) is a potent inductor of the inflammatory response in innate immune response [3]. Anti-TNF-α mAbs are undoubtedly to be clinically efficacious for autoimmune diseases, especially for RA. [4]. Despite given the profound therapeutic potential of anti-TNF-α drugs, around 30% of patients are refractory to anti-TNF-α drugs, responses are frequently considered partial in patients who respond. The interaction of TNF-α receptor (TNF-α R) and TNF-α activates two signal pathways: IKK/NF-κB and MAPK/AP-1 pathways. Activated pathways, in turn, trigger the production of inflammatory cytokines and chemokines. These TNF-induced cytokines are involved in the initiation and progression of inflammatory diseases. Recent studies indicate that there exists a shared cytokine framework, including TNF-α, interleukin (IL)-1, IL-6, IL-17 and IL-23, in certain inflammatory diseases [5]. TNF-α correlates to promote either chemokines (such as IL-8 and monocyte chemotactic protein 1) or angiogenesis [3]. Blockage of IL-1, IL-6, IL-17, or IL-23 has shown clinical efficacy in RA patients [6].

IL-17 is one of main inflammatory cytokines that are secreted by the Th17 T helper cell subset. It is an important contributor to the pathogenesis of autoimmune diseases including RA [7]. When IL-17 binds to its receptor, the activation of NF-κB and MAPKs signaling pathways are subsequently induced. The IL-17 signaling contributes to the inflammatory response that causes cartilage destruction [8]. IL-17 plays a key role in inducing the production of TNF-α or other pro-inflammatory cytokines in synovial cells and chondrocytes of RA patients [9]. Anti-IL-17 treatment is effective in reducing the onset of clinical arthritis symptoms [10]. Dual inhibition of IL-1α/β [11], IL-1β/IL-17 [12], TNF-α/IL-17 [13, 14], TNF-α/IL-6R [15] or TNF-α/Angiopoietin2 [16] is more potent than single targeting for the treatment of RA and other inflammatory disorders.

Bispecific antibodies (bsAbs) binding two different antigens can overcome limitations of monotherapies due to their beneficial characteristics [17-20]. However, the most formats of bsAb, such as or dual-variable domain (DVD) IgG, may result in the potential immunogenicity because of incorporated artificial linkers. Other factors, such as low expression, poor stability, poor affinity and poor biocompatibility, frequently hinder the pharmaceutical development of bsAbs [21]. Here, we generated a stable IgG-like bsAb which kept the properties of intact IgG molecules. This bsAb has not only functional characteristics of the anti-TNF-α antibody, but also additional activity that neutralizes IL-17A. It significantly suppressed the inflammatory cytokine response by simultaneously neutralizing TNF-α and IL-17A in RA models. These studies support that combination therapies for the treatment of RA or other inflammatory disorder are highly promising approaches.

## RESULTS

### Generation of the native-like bsAb through electrostatic Fc pairing

**Figure 1:**
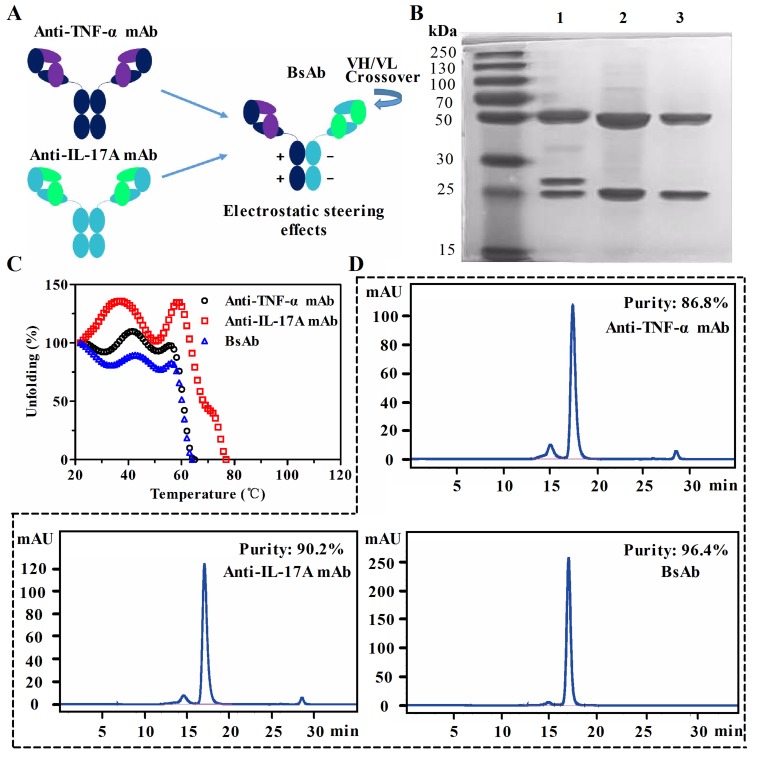
Generation and characterization of anti-TNF-α/IL-17A bispecific antibody (bsAb) **A.** Schematic diagram of bsAb generated by fusion of anti-TNF-α antibody and anti-IL-17A antibody. BsAb consists of a crossed anti-IL-17A arm (CH1-C exchange) and an uncrossed anti-TNF-α arm. The parental monoclonal antibodies (mAb) are shown on the left. Fc parts of heavy chains are paired through an electrostatic steering effects. **B.** SDS-PAGE analysis of purified bsAb (lane 1), anti-IL-17A mAb (lane 2), and anti-TNF-α mAb (lane 3). **C.** Comparison of thermal stability of three antibodies by circular dichroism. **D.** The purity analysis of bsAb and parental mAbs by a size exclusion chromatography column.

To generate a native-like bispecific molecule, it was built based on a 1+1 of two half IgG. Heterogenous heavy chain (HC) Fc regions were paired through electrostatic steering effects. As shown in Figure 1A, two pairs of oppositely charged residues (K392-D399 and D399-K409) are used in the CH3-CH3 domain. The mutations of K409D and K392D in the first Fc, D399K and E356K in the second Fc resulted in the pairing of two heterogenous heavy chains by altering the polarity. The correct association of the light chain and their cognate heavy chains was achieved by exchanging the variable heavy (VH) and the variable light (VL) domains within the anti-IL-17A antibody.

Co-transfection of HEK293 suspension cells with expression vectors encoding the anti-TNF-α/IL-17A bsAb resulted in the production of soluble proteins with the high purity in SDS-PAGE assays (Figure 1B). Yields of anti-TNF-α mAb and bsAb were almost the same (∼ 7.6 mg/L), and the yield of anti-IL-17A mAb was about 10 mg/L (Supplementary Table S1). Because of exchange of the VH and VL in bsAb, one of the light chain of the antibody migrated to a position corresponding to a higher molecular weight. The thermal stability of bsAb was compared with that of the parental mAbs. As shown in Figure 1C, the melting temperature (Tm) of bsAb was lower than that of anti-IL-17A mAb (Tm = 78°C), but is equivalent to that of the anti-TNF-α mAb (Tm = 68°C). Purities of the antibodies were analyzed by a size exclusion chromatography column (SEC) (Figure 1D). Monomer portions of the anti-TNF-α antibody, anti-IL-17A antibody, and bsAb were 86.8%, 90.2%, and 96.4%, respectively. These data suggest that the generated bsAb through Fc heterodimerization retain physical properties of parental mAbs.

### Binding abilities of bsAb to TNF-α and IL-17A

**Figure 2:**
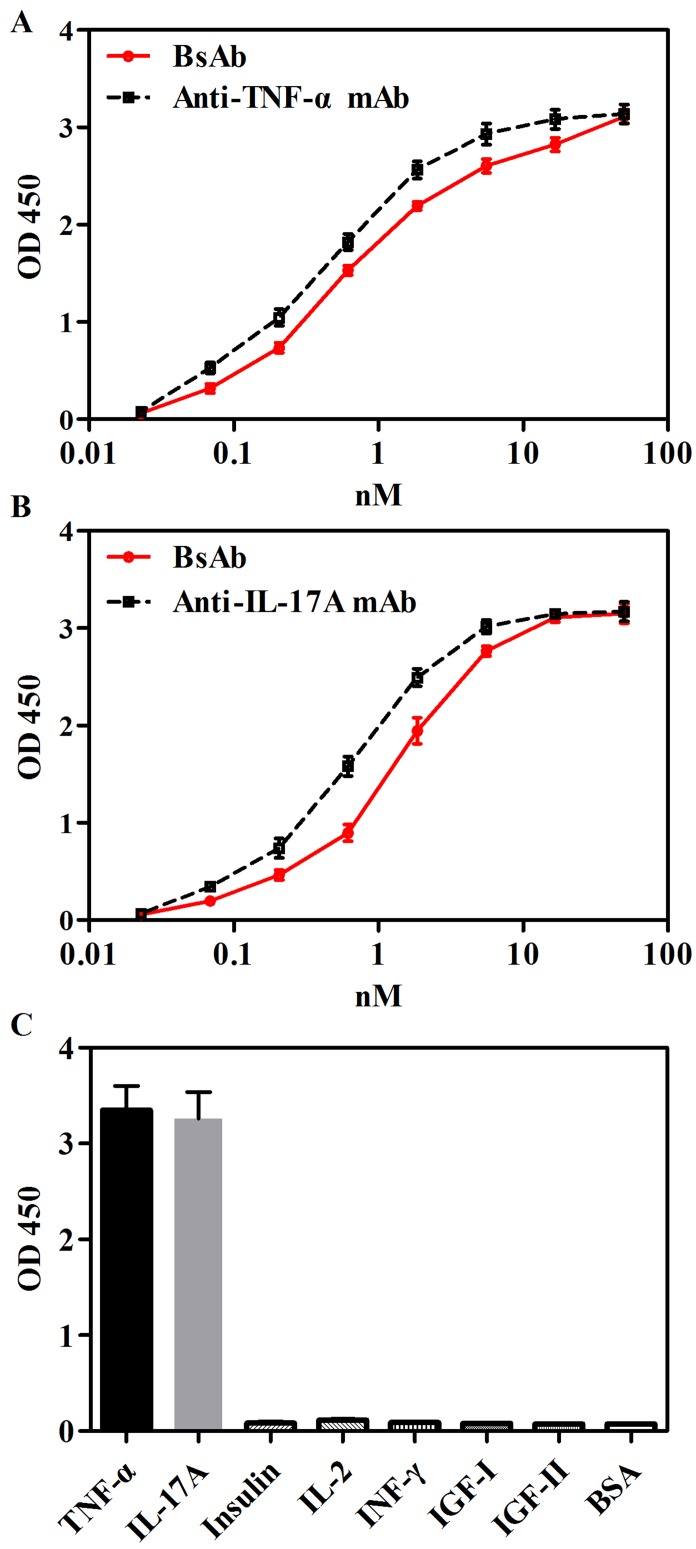
Binding abilities of bsAb to TNF-α and IL-17A in ELISA **A.** Immobilized TNF-α was bound with anti-TNF-α mAb or bsAb with serial dilutions. **B.** Immobilized IL-17A was bound with anti-IL-17A mAb or bsAb with serial dilutions. **C.** Specificity of bsAb were measure by detecting seven antigens, including TNF-α, IL-17A, insulin, IL-2, IGF-I, IGF-II, and INF-γ. Bound antibodies were detected with a horseradish peroxidase-conjugated anti-human Fc antibody and measured as optical densities (OD) at 450nm.The data are a representative of 3 separate experiments.

The binding of bsAb to TNF-α and IL-17A was measured by ELISA. As shown in Figure 2A and 2B, EC50 values of bsAb binding to IL-17A and TNF-α were comparable to parental anti-IL-17A and anti-TNF-α mAbs. The specificity of bsAb was detected against seven antigens, including TNF-α, IL-17A, insulin, IL-2, insulin-like growth factor (IGF)-I, IGF-II, and Interferon (INF)-γ by ELISA. BsAb bound to targets TNF-α and IL-17A, but not other irrelevant antigens (Figure 2C).

**Figure 3:**
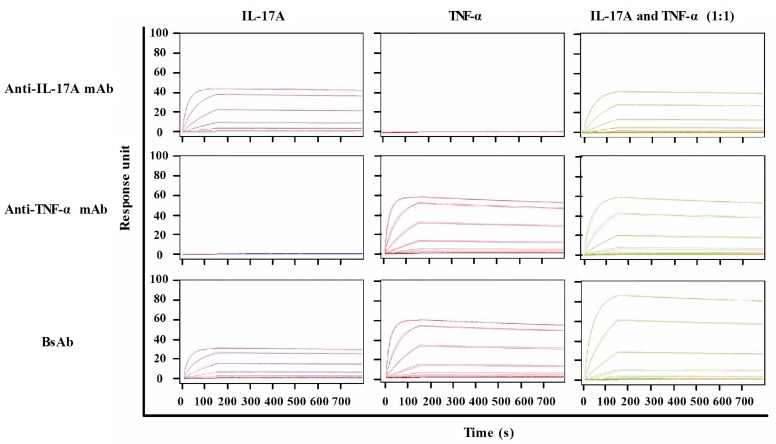
SPR analysis of antibodies against TNF-α and IL-17A Anti-IL-17A mAb, ant-TNF-α mAb, or bispecific antibody was captured by anti-human Fc antibody on to a CM5 chip. A range of TNF-α (0 nM to 100 nM) and IL-17A (0 nM to 100 nM) were injected over all antibodies. Data was fitted to a 1:1 Langmuir binding model to determine binding parameters.

Surface plasmon resonance (SPR) demonstrated the functionality of both binding moieties toward the respective antigens. To measure the binding kinetics of antibodies to corresponding ligands, TNF-α, IL-17A or their mixture with different concentrations was flowed over fixed antibodies on a CM5 chip *via* an anti-human Fc antibody. SPR sensorgrams were presented in Figure 3. The kinetics values of all antibodies were summarized in Table 1. The calculated association rate constant (kon: 4. 7 × 10^5^ (mol/L)^-1^s^-1^) and the dissociation rate constant (koff: 1.6 × 10^-5^ s^-1^) of bsAb binding to TNF-α were similar to that of anti-TNF-α antibody (kon: 4.7 × 10^5^ (mol/L)^-1^s^-1^; koff: 1.7 × 10^-5^ s^-1^). Similarly, the binding affinity of bsAb to TNF-α (K_D_: 0.33 nmol/L) was comparable to that of anti-TNF-α antibody (K_D_: 0.37 nmol/L). BsAb bound to immobilized IL-17A with a high affinity (K_D_: 0.2 nmol/L), which is similar to anti-IL-17A antibody (K_D_: 0.17 nmol/L). Simultaneous binding to IL-17A and TNF-α was also shown. A mixture of TNF-α and IL-17A was used to flow over to determine maximum responses. The Rmax/capture of bsAb to the mixture was apparently higher than that of parental mAbs. It suggests that a bivalent bsAb could enhance avidity effects better than monovalent reagent.

**Table 1:** SPR summary of anti-TNF-α/IL-17A bsAb, anti-TNF-α mAb and anti-IL-17A mAb.

Antibody	Antigen	kon, (mol/L^-1^)s^-1^	koff, s^-1^	Rmax	K_D_, mol/L
Anti-IL-17A mAb	IL-17A	4.2×10^5^	7.0×10^-5^	43.6	1.7×10^-10^
	TNF-α	No Binding			
	IL-17A+TNF-α	4.4×10^5^	7.5×10^-5^	43.1	1.7×10^-10^
Anti-TNF-α mAb	IL-17A	No binding			
	TNF-α	4.7×10^5^	1.7×10^-4^	57.8	3.7×10^-10^
	IL-17A+TNF-α	4.7×10^5^	1.7×10^-4^	60.3	3.7×10^-10^
BsAb	IL-17A	4.0×10^5^	7.9×10^-5^	30.6	2.0×10^-10^
	TNF-α	4.7×10^5^	1.6×10^-4^	59.1	3.3×10^-10^
	IL-17A+TNF-α	4.6×10^5^	1.2×10^-4^	88.6	2.5×10^-10^

### Neutralization of TNF-α-mediated cellular cytotoxicity by bsAb

**Figure 4:**
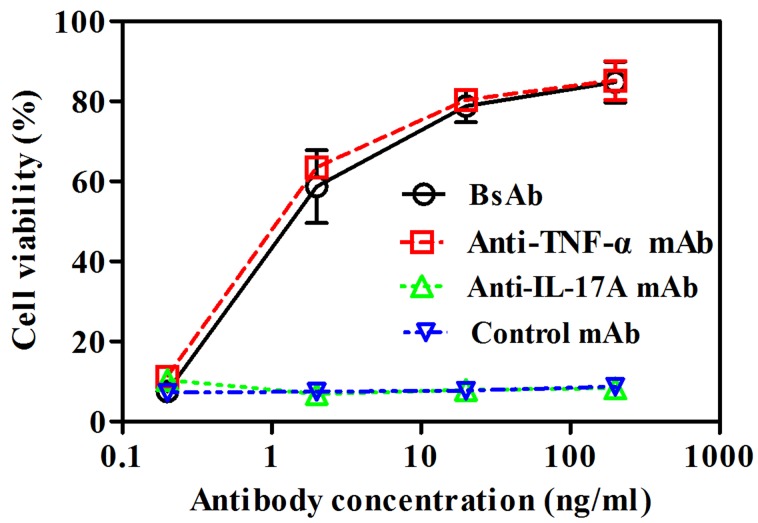
Neutralization of TNF-α-mediated cellular cytotoxicity by bsAb L929 cells were incubated with 1 ng/ml of TNF-α and 4 μg/ml of actinomycin D. Then, cells were treated with various concentrations of bsAb, anti-TNFα mAb, anti-IL-17A mAb and anti-IGF-I/II mAb m708.5 as a control antibody. After 24 h, cell viabilities were determined by MTT method. The error bars represent mean ± SEM. Data shown are representatives of three independent experiments.

Fibroblast L929 cell line has been used for the standard analysis of TNF-α cytotoxicity [22]. We employed assays to assess the ability to prevent TNF-α-mediated cell cytotoxicity. BsAb inhibited TNF-α-induced L929 cell cytotoxicity in a dose-dependent manner, which was comparable to that of anti-TNF-α mAb (Figure 4). Anti-IL-17A mAb and control anti-IGF-I/II mAb m708.5 had no effect on neutralization of TNF-α.

### Addition and synergy of combined IL-17A and TNF-α to induce the inflammatory cytokine responses on FLS

**Figure 5:**
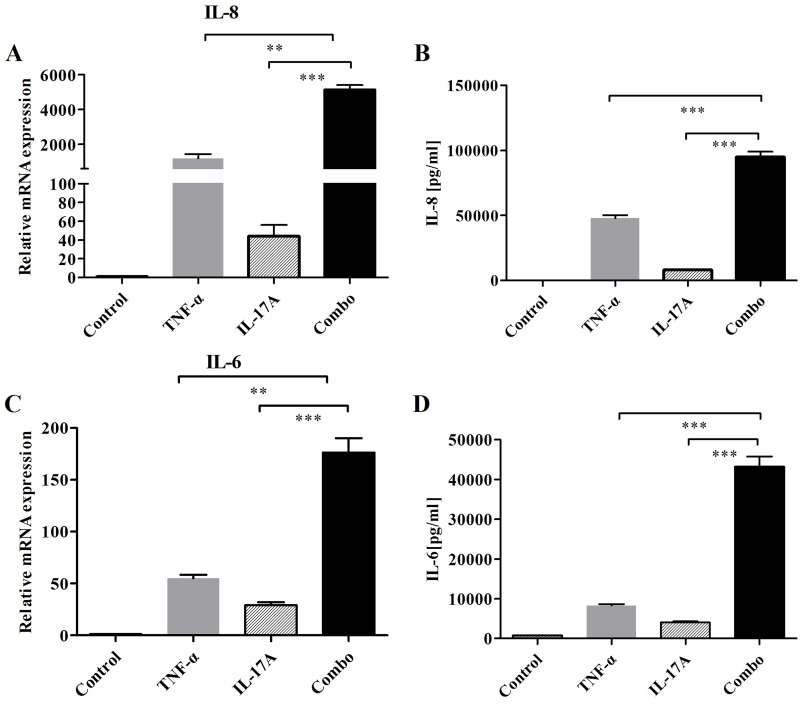
Addition and synergy of combined IL-17 and TNF-α to induce the production of IL-6 and IL-8 on FLS **A.** After cells were stimulated with IL-17 and TNF-α for 12 h, mRNA expressions of cytokines (IL-6, IL-8) were determined by realtime-PCR. **B.** Secretions of cytokines (IL-6 and IL-8) in culture media were measured by ELISA. All results are representative of at least three independent experiments.

FLS is a crucial resident cell type involved in RA pathogenesis. Here, we investigated the production of key inflammatory cytokines in RA-FLS after co-treatment with IL-17A and TNF-α. When RA-FLS cells were incubated with IL-17A, TNF-α, or both, mRNA expressions of IL-6 and IL-8 were assessed. In addition, secretions of cytokines (IL-6 and IL-8) in culture media were measured by ELISA. Combination of IL-17A and TNF-α significantly resulted in additive or synergistic increase of IL-6 and IL-8 expressions (Figure 5). Thus, IL-17A appears to exert an additive and synergistic effect with TNF-α on FLS. The above results suggest that FLS is an effective model to assess combined blockade of bsAbs in next studies.

### Inhibition of bsAb on the production of neutrophil and Th17 chemokines on FLS

**Figure 6:**
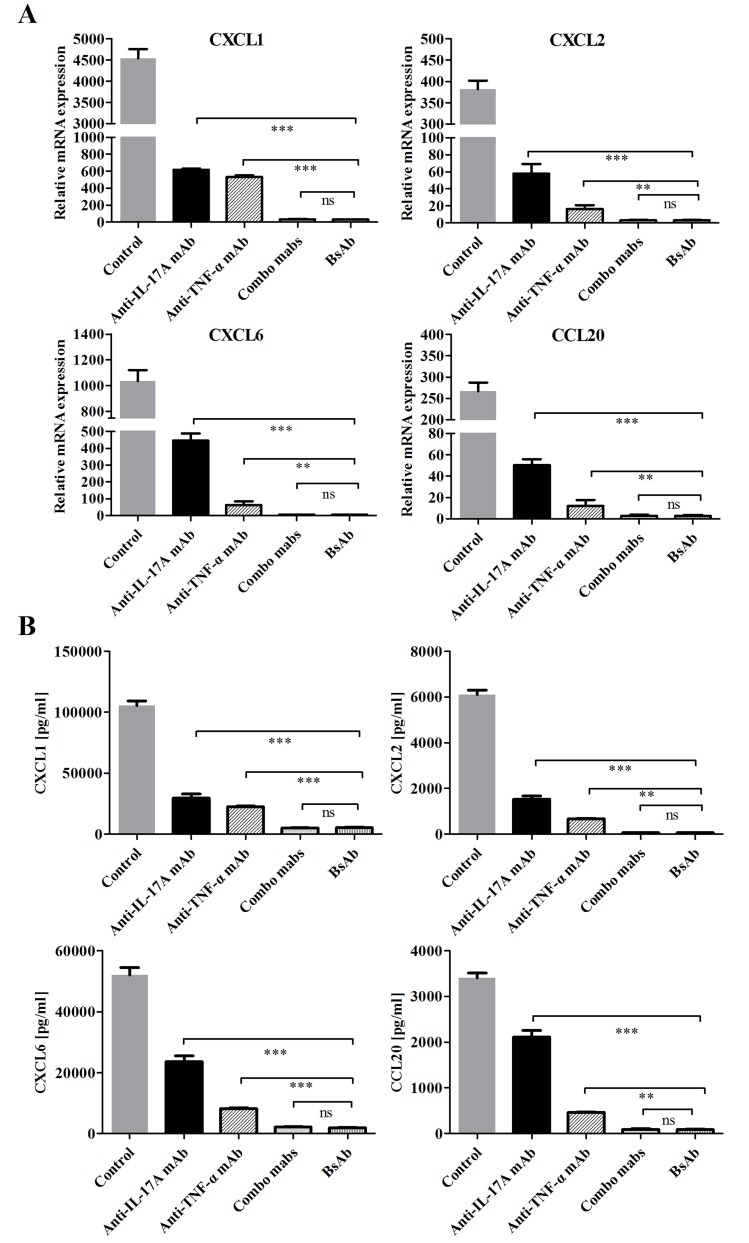
Inhibition of bsAb on the production of neutrophil and Th17 chemokines on FLS **A.** FLS cells were incubated with IL-17 and TNF-α or in the presence of different, antibodies. The mRNA expression levels of CXCL1, CXCL2, CXCL6, and CCL20 were measured by realtime-PCR after stimulation for 12 h. **B.** After FLS were treated, the secretion of chemokines from RA-FLS were measured by ELISA. All results are representative of at least three independent experiments.

Neutrophil recruitment can be promoted in inflammation sites through FLS activation by IL-17A and TNF-α [23, 24]. Gene expression levels of mediator cytokines were very low when IL-17A and TNF-α were used alone, but combination of two cytokines synergistically increased chemokine expression. We evaluated the inhibitory effects of bsAb on the production of inflammatory mediators on FLS. The mRNA and protein expressions of neutrophil chemokines (CXCL1, CXCL2, CXCL6) and the Th17 chemokine (CCL20) were significantly decreased when both IL-17A and TNF-α were blocked (Figure 6). BsAb showed a superior effect to single treatment and comparable of the combination of two mAbs. Our results suggest that blocking two antigens simultaneously is better than inhibition of solo antigen.

### Inhibition of inflammatory cytokine responses in RA-FLS by bsAb against TNF-α and IL-17A

**Figure 7:**
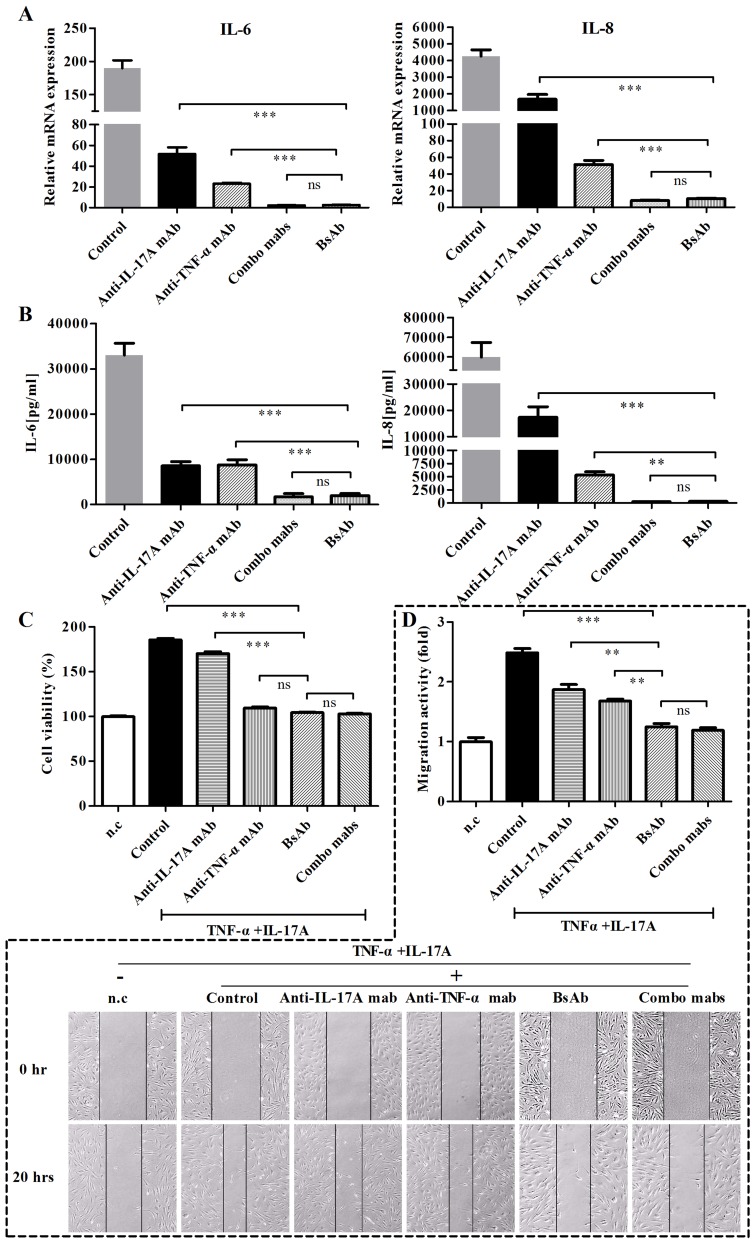
Inhibition of bsAb on the production of IL-6 and IL- 8 and proliferation, and migration of FLS FLS cells were stimulated with TNF-α and IL-17A or in the presence of different, antibodies. **A.** The mRNA levels of IL-6 and IL- 8 were determined by realtime-PCR. **B.** Secretions of cytokines (IL-6 and IL-8) in culture media were measured by ELISA. **C.** Anti-proliferative effects of bsAb on FLS. After FLS cells were incubated with TNF-α and IL-17A, antibodies was added during the incubation. Cell proliferation was assessed using MTT assay. **D.** The anti-migration effects of bsAb on FLS were evaluated by the scratch assay. After wounds were formed, FLS were incubated with antibodies in the presence of TNF-α and IL-17A. Parallel lines represent the borders of the wounds.

The gene levels of IL-6 and IL-8 were examined in FLS from RA patients after FLS were treated with antibodies (Figure 7A). Anti-TNF-α or anti-IL-17A mAbs had effects to prevent cytokine induction in FLS stimulated with both cytokines in the mRNA level. BsAb showed superior inhibitory effects than single mAb treatments. Meanwhile, we detected the secretion levels of IL-6 and IL-8 in cell supernatants by ELISA (Figure 7B). Consistent results were seen after treatment with bsAb or combination of two mAbs. The secretion of IL-6 and IL-8 was significantly decreased by bsAb. Thus, dual blockage of TNF-α and IL-17A cytokines could sustain relatively low levels of IL-6 and IL-8 responses.

### Anti-proliferative and anti-migration effect of bsAb on FLS

The anti-proliferative effects of bsAb were analyzed on FLS *in vitro*. BsAb and anti-TNF-α mAb inhibited the proliferation of FLS whereas anti-17A mAb did not (Figure 7C). We predict that TNF-α is a key contributor for the proliferation of synoviocytes. Because TNF-α and IL-17 are both involved in the migration of fibroblasts [25], the scratch assay could be used to examine the anti-migration function of the bsAb. The co-treatment of IL-17A and TNF-α significantly increased the cell migration activity. All antibodies could inhibit the migration of RA-FLS in different degrees. The migration activity of FLS cells appeared to be the lowest when both the antigens were blocked by bsAb simultaneously (Figure 7D).

## DISCUSSION

Though TNF-α and IL-17A are independently involved in pathophysiology of RA, elevated IL-17 and TNF-α appear in the synovium of RA patients [26]. They act in synergy as inducing mediators of inflammation in fibroblast-like synoviocytes. Combined blockade of TNF-α and IL-17A pathways results in decrease of arthritic symptoms in patients who fail to respond to single-cytokine inhibition [27]. Inhibitions are believed to be selective because normal immunity would be not interfered.

We generated a native-like bsAb through a combination method of electrostatic Fc pairing and light chain crossover. It is different with other reported anti-TNF/IL-17A bsAbs. BsAb is based on fully human anti-TNF-α and IL-17A antibodies. The electrostatic pairing results in less mismatching of heavy chains than the knob-into-hole pairing. Mismatched heavy or light chains are main problems during the production of IgG-like bsAbs. Importantly, it aborts to use any artificial linkers that induce possible secondary adverse effects in body. In SPR assay, bsAb showed high-affinities to human TNF-α and IL-17A. It partially bound to mouse TNF-α and did not respond to mouse IL-17A (Supplementary Figure S1). It is still challenging to test bioactivitie of anti-human IL-17A antibodies in RA mouse models. We observed that bsAb obviously enhanced response of captured antigens when it bound two antigens. It is a unique feature of bsAb so that soluble polypeptides can be quickly eliminated through the bivalent binding [18, 20].

Several crucial factors CXCL1, CXCL2, CXCL5, CXCL6 and IL-8 are involved in the recruitment of neutrophils in inflammation of RA [15, 28]. Th17 cells are known to contribute to arthritis pathogenesis in some patients treated with anti-TNF drugs [29]. FLS are key effector cells in rheumatoid arthritis. Targeting FLS might improve therapy effects in inflammatory arthritis without inhibiting systemic immunity [30]. Anti-TNF-α/IL-17A bsAb significantly inhibited the expression levels of neutrophil chemokines CXCL1, CXCL2, CXCL6 and the Th17 chemokine secreted by FLS. IL-17A is a major inducer of IL-6 and IL-8 that are key pro-inflammatory cytokines in inflammation and joint damage of RA [31]. We observed that IL-6 and IL-8 were abundantly secreted by IL-17A- and TNF-α-stimulated FLS. The observation is consistent with previous reports [26, 32]. Though the bsAb showed similar inhibition effects with two monospecific reagents, bsAb would be less costly than two monospecific antibodies and leads to less immunogenic response in patients [33].

In conclusion, we generated a native-like bsAb that has the potential as therapeutic molecules in the treatment of inflammatory diseases. Dual inhibition of TNF-α/IL-17 is efficacious to reduce signaling redundancy in rheumatoid arthritis. It is interesting to further investigate the efficacy and safety of bsAb in humanized RA transgenic mouse models.

## MATERIALS AND METHODS

### Cells and cell culture

Fibroblast cell line L929 was purchased from the Type Culture Collection of the Chinese Academy of Sciences, Shanghai, China. Fibroblast-like synoviocytes (FLS) were isolated from synovial tissue of a RA patient. All procedures followed were in accordance with the ethical standards approved by Jilin University. FLS cells were cultured in RPMI1640 medium (Hyclone) containing 10% FBS, 100 U/ml penicillin, and 100 μg/ml streptomycin. L929 cells were cultured in DMEM medium (Hyclone) containing 10% fetal bovine serum (FBS) (Hyclone). All the cells used in the experiment were in passage 4 to 10.

### Construction of antibody expression vectors

Antibody variable region sequences of anti-TNF-α (adalimumab) mAb and anti-IL-17A (secukinumab) mAb were used to make IgG or bsAb expression vectors. As shown in Figure 1A, anti-TNF-α/IL-17A bispecific antibodies were generated based on these antibodies (human IgG1 backbone). The Fc regions of two heavy chains were paired by an electrostatic steering effects methodology [34]. Sequentially, correct association of the light chains and their heavy chains was achieved by exchange of variable heavy (VH) and variable light (VL) domains within the antigen binding fragment (Fab) of anti-IL-17A of bsAb. Meanwhile, IgGs of anti-TNF-α and anti-IL-17A were made in the same backbone. All genes were synthesized by Genescript.

### Expression and purification of antibodies

Serum-free suspension HEK293 cells (Life Technologies) were used to express bsAb and mAbs. Transfection into HEK293 cells was performed according to the instruction as described previously [19]. Briefly, HEK293 cells were cultivated in FreeStyle™ 293 Expression Medium (Life Technologies). The mixture of expression plasmids at concentration of 10 μg/ml and PEI (25000 MW, Polysciences) at final concentration of 20 μg/ml was prepared in 1/10 volume of fresh culture medium. After incubation for 15 min at room temperature, the mixture was slowly dropped into cells. After four days of post-transfection, the culture supernatant was harvested. Antibodies were purified from cell culture supernatants by the Protein A Sepharose TM chromatography (GE Healthcare).

### Size-exclusion chromatography

Antibodies were added to a Sepax SRT SEC-500 column (Sepax Technologies Inc.,) respectively. And the composition of the mobile phase was 150 mM sodium phosphate at a pH of 7.2. The flow rate was 1.0 ml/min and we used 280 nm detection wavelength to monitor the eluent.

### Circular dichroism

Far UV circular dichroism spectra were recorded on a Jasco J-815 spectropolarimeter (Jasco International). Antibodies were prepared in 1 ml of 20 mM phosphate buffer containing 0.15 M NaCl at pH 7.0. The spectra were analyzed by monitoring the molar ellipticity changes at 222 nm as a function of temperature increase. The sample was heated at a rate of 10 °C/min over a temperature range of 20-80°C. The normalized ellipticity signal was plotted as a function of temperature to understand changes in thermal stability.

### ELISA assay

TNF-α and IL-17A (Peprotech) were coated on 96-well ELISA plates overnight at 4°C. Antibodies with different dilutions were incubated for 1 h. Bound antibodies were detected with the secondary anti-human Fc-HRP antibody (1:2,000 dilution; Life Technologies). The 2, 20-azino-bis-(3-ethylbenzthiazoline-6-sulfonic acid) substrate (Sigma) was added and the reaction was read at 450 nm.

### Surface plasmon resonance

Interactions between antibodies and TNF-α and IL-17A were analyzed by SPR technology using a Biacore X100 instrument (GE healthcare). Anti-TNF-α mAb, anti-IL-17A mAb or bsAb was immobilized to the surface of a CM5 sensor chip using the standard amine-coupling chemistry method. For analysis of the kinetics of interactions, varying concentrations of TNF-α (0 nM to 100 nM at three-fold serial dilutions) IL-17A(0 nM to 100 nM at three-fold serial dilutions), or a mixture of both TNF-α and IL-17A(0 nM to 50 nM at three-fold serial dilutions) were injected at flow rate of 30 μl/min using running buffer HBS-EP containing 10 mmol/L HEPES, 150 mmol/L NaCl, 3 mmol/L EDTA, and 0.05% Surfactant P-20 at pH7.4. Binding data were analyzed using a 1:1 Langmuir model. Langmuir binding model to obtain the association rate constant (ka), dissociation rate constant (kd) and the equilibrium dissociation rate constant (K_D_). All the experiments were done at 25°C.

### Cell cytotoxicity assay

L929 cells were cultured in 96-well plates at 3.5 × 10^4^ cells/well in 100 μl of medium supplemented with 10% FBS overnight, and then incubated with 1 ng/ml TNF-α plus 4 μg/ml of Actinomycin D in the presence of various concentrations of antibodies for 24 h at 37°C. Cell viabilities were determined by adding 10 μl of (3-(4,5-dimethyl-2-thiazolyl)-2,5-diphenyl-2-H-tetrazolium bromide (Promega). Absorbance was measured at 450 nm using a Biotek ELISA plate reader.

### Real-time PCR

FLS cells were seeded in 6-well plates (5×10^4^ cells/well), and incubated for two days. After removing medium, cells were incubated with mixtures of TNF-α (0.5 ng/ml) and IL-17A (50 ng/ml). Inhibition assays were performed by adding 100 μg/ml of the single mAb, bsAbs, or the mixture of two mAbs (50 μg/ ml+50 μg/ml). After 12 h, total RNA was extracted from cells using the trizol method and then transcribed to complementary DNA using an AffinityScript QPCR cDNA Synthesis Kit (Agilent). Quantitative PCR was performed using an Mx3000P Real-Time PCR System (Agilent). The resulting amplification and melting curves were analyzed to ensure specific PCR product. Ct values were used to calculate the fold change in transcript levels.

### Detection of cytokines and chemokines by ELISA

FLS cells were seeded in 6-well plates (5×10^4^ cells/well), and the plates were cultured for two days. After removing medium, cells were incubated with mixtures of TNF-α (0.5 ng/ml) and IL-17A (50 ng/ml). Inhibition assays were performed by 100 μg/ml of the single mAb, bsAbs, or the mixture of two mAbs (50 μg/ml+50 μg/ml). After 24 h, the secreted IL-6 and IL-8 in culture supernatants were measured using ELISA kits (eBioscience) according to the manufacturers’ instructions.

### Cell proliferation assay

FLS cells were incubated with or without IL-17A (100 ng/ml), and human TNF-α (20 ng/ml) for 48 h. During the incubation, cells were treated with 100 μg/ml of the single mAb, bsAbs, or the mixture of two mAbs (50 μg/ml+50 μg/ml). FLS were also cultured without cytokines and antibodies as a negative control. Cell viabilities were determined by adding 10 μl of (3-(4,5-dimethyl-2-thiazolyl)-2,5-diphenyl-2-H-tetrazolium bromide (Promega). Absorbance was measured at 450 nm using a Biotek ELISA plate reader.

### Scratch assay

FLS cells were seeded in 6-well plates (5×10^4^ cells/well). When the confluence of FLS reached 100%, the cell layer was scratched with a pipette tip. After washed with medium, the cells were cultured with fresh medium. The mixture of TNF-α (20 ng/ml) and IL-17A (200 ng/ml) were added to cell wells. During the cell culture, FLS cells were treated with 100 μg/ml mAb, bsAbs or the mixture of the two mAbs (50 μg/ml+50 μg/ml). The images were acquired by inverted microscope at 0 and 20 h. The migration activities were calculated as follows: migration activity = 1- (wounded area at 20 h) / (wounded area at 0 h).

### Statistical analysis

Mean ± SE values were calculated, and the significance of differences was calculated using the Prism Graphpad software. *P* values less than 0.05 were considered significant.

## SUPPLEMENTARY MATERIALS FIGURE AND TABLE



## Abbreviations

(TNF): tumor necrosis factor
(RA): rheumatoid arthritis
(bsAb): bispecific antibodiy fibroblast-like synoviocytes
(mAb): monoclonal antibody
(TNF-α R): TNF-α receptor
(DVD): dual-variable domain
(HC): heavy chain
(VH): variable heavy
(VL): variable light
(SEC): size exclusion chromatography column
(SPR): Surface plasmon resonance
(FBS): fetal bovine serum
(Tm): melting temperature
