# Supplementary material for: A native-like bispecific antibody suppresses the inflammatory cytokine response by simultaneously neutralizing tumor necrosis factor-alpha and interleukin-17A
Source: Oncotarget. 2017 Aug 3;8(47):81860–72. doi: 10.18632/oncotarget.19899 (PMC5669854; doi:10.18632/oncotarget.19899)
Supplement: Supplementary file 1 [file oncotarget-08-81860-s001.docx]

**Supplementary Table S1:** The antibody expression of anti-IL-17A mAb, anti-TNF-α mAb and bsAb.

| Antibody | Anti-IL-17A mAb | Anti-TNF-α mAb | BsAb |
| --- | --- | --- | --- |
| Expression volume | 200 ml | 200 ml | 200 ml |
| Plasmid | 200 μg | 200 μg | 200 μg |
| Expression level | 10.2mg/L | 7.64mg/L | 7.57mg/L |
| Total amount | 2.04mg | 1.53mg | 1.51mg |


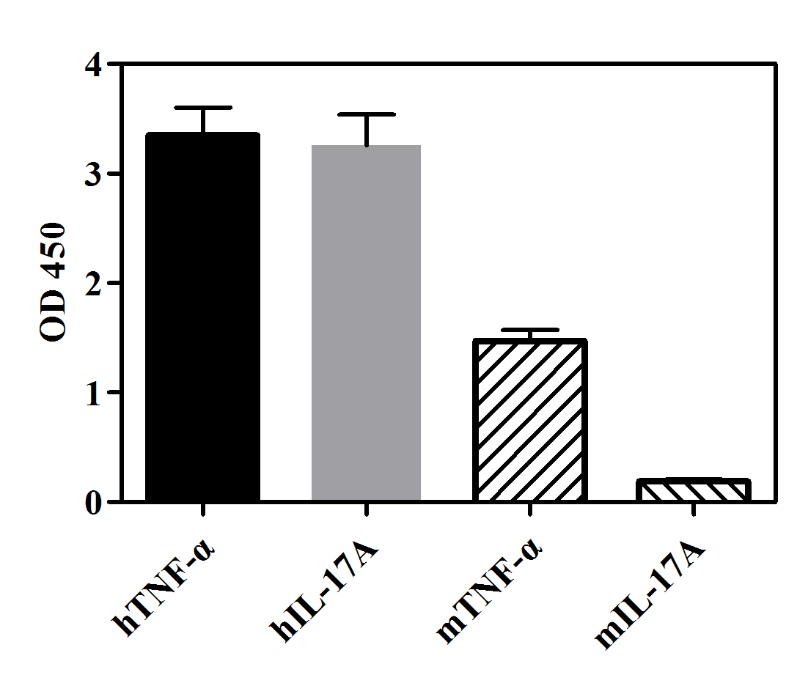


**Supplementary Fig.S1. Binding of bsAb against human/mouse TNF-α and IL-17A in ELISA.**
